# Mapping Monkeypox Transmission Risk through Time and Space in the Congo Basin

**DOI:** 10.1371/journal.pone.0074816

**Published:** 2013-09-05

**Authors:** Yoshinori Nakazawa, R. Ryan Lash, Darin S. Carroll, Inger K. Damon, Kevin L. Karem, Mary G. Reynolds, Jorge E. Osorio, Tonie E. Rocke, Jean M. Malekani, Jean-Jacques Muyembe, Pierre Formenty, A. Townsend Peterson

**Affiliations:** 1 Centers for Disease Control and Prevention, Atlanta, Georgia, United States of America; 2 Oak Ridge Institute for Science and Education, Oak Ridge, Tennessee, United States of America; 3 Department of Pathological Sciences, School of Veterinary Medicine, University of Wisconsin, Madison, Wisconsin, United States of America; 4 USGS National Wildlife Health Center, Madison, Wisconsin, United States of America; 5 University of Kinshasa, Kinshasa, Democratic Republic of Congo; 6 Institut National de Recherche Biomédicale, Kinshasa, Democratic Republic of Congo; 7 Department of Communicable Diseases Surveillance and Response, World Health Organization, Geneva, Switzerland; 8 Biodiversity Institute, University of Kansas, Lawrence, Kansas, United States of America; Tulane University School of Public Health and Tropical Medicine, United States of America

## Abstract

Monkeypox is a major public health concern in the Congo Basin area, with changing patterns of human case occurrences reported in recent years. Whether this trend results from better surveillance and detection methods, reduced proportions of vaccinated vs. non-vaccinated human populations, or changing environmental conditions remains unclear. Our objective is to examine potential correlations between environment and transmission of monkeypox events in the Congo Basin. We created ecological niche models based on human cases reported in the Congo Basin by the World Health Organization at the end of the smallpox eradication campaign, in relation to remotely-sensed Normalized Difference Vegetation Index datasets from the same time period. These models predicted independent spatial subsets of monkeypox occurrences with high confidence; models were then projected onto parallel environmental datasets for the 2000s to create present-day monkeypox suitability maps. Recent trends in human monkeypox infection are associated with broad environmental changes across the Congo Basin. Our results demonstrate that ecological niche models provide useful tools for identification of areas suitable for transmission, even for poorly-known diseases like monkeypox.

## Introduction

Monkeypox virus (MPXV) was first isolated from captive cynomolgus monkeys (*Macaca fascicularis*) in Denmark in 1959 [1]; in subsequent years, additional monkeypox (MPX) cases were reported in captive non-human primates from research facilities and zoos [2]. In 1970, the first human MPX case was described from Basankusu, Democratic Republic of Congo (DRC) [3]. In humans, the symptoms and progress of this disease are very similar to those presented by smallpox, albeit with lower fatality rates. MPXV belongs to the genus *Orthopoxvirus*, and as such shares cross-immunological protection with other members of the genus, including *vaccinia virus*, the agent used in the smallpox vaccine. Since worldwide eradication of smallpox, routine smallpox vaccination programs were terminated, increasing over time the portion of the human population potentially at risk for MPXV infection. As a result, monkeypox has recently been identified as an important emerging disease, raising public health concerns [4], [5], [6].

Increased numbers of human MPX cases reported in recent years compared to those reported at the close of the smallpox era may stem from environmental, anthropogenic, or technological factors. MPX transmission to humans has been shown to be associated with particular environmental conditions [7], so environmental changes could alter the locations of areas suitable for transmission, and new cases of the disease could appear in areas where it did not previously occur; other areas formerly suitable for transmission may see reduced transmission. Human population growth and human activities may be associated with increasing human contact with possible wildlife reservoirs of MPXV, facilitating transmission of the virus to people [8], [9]. Finally, concentrating efforts and resources for the study of MPX in smaller geographic areas, in conjunction with the use of streamlined diagnostic techniques, such as real time-PCR [10], may complicate comparisons of MPX prevalence between the present and the past, compromising the reliability of inferences derived from such comparisons.

Ecological niche modeling (ENM) approaches have been used amply in biogeography, ecology, and macroecology in the last 15 years [11], [12], [13], [14], and increasingly have been used to characterize the geography of disease transmission [15], [16], [17], [18], [19], [20], [21], [22], [23]. In the case of monkeypox, previous analyses [7], [24], [25] have used ENM algorithms to produce maps of monkeypox transmission potential in central and western Africa. These models attempt to delimit potential suitable areas for human monkeypox transmission based on correlations between recorded localities of human cases and particular environmental conditions [7], although the details of transmission of MPXV in wildlife remain poorly understood [4], [26].

Here, we explore further and in greater detail the association between environmental conditions and human MPX cases, with the specific purpose of mapping changing areas suitable for transmission across the Congo Basin. Specifically, we (1) build environmental datasets that match closely the temporal characteristics of reported cases, (2) create and test rigorously ENMs based on these improved datasets, and (3) project the models onto more recent environmental conditions to evaluate their capacity to anticipate future transmission areas. The result is an improved picture of spatial variation in suitable areas for MPXV transmission across the Congo Basin through time.

## Methods

### Human case data

**Figure 1:**
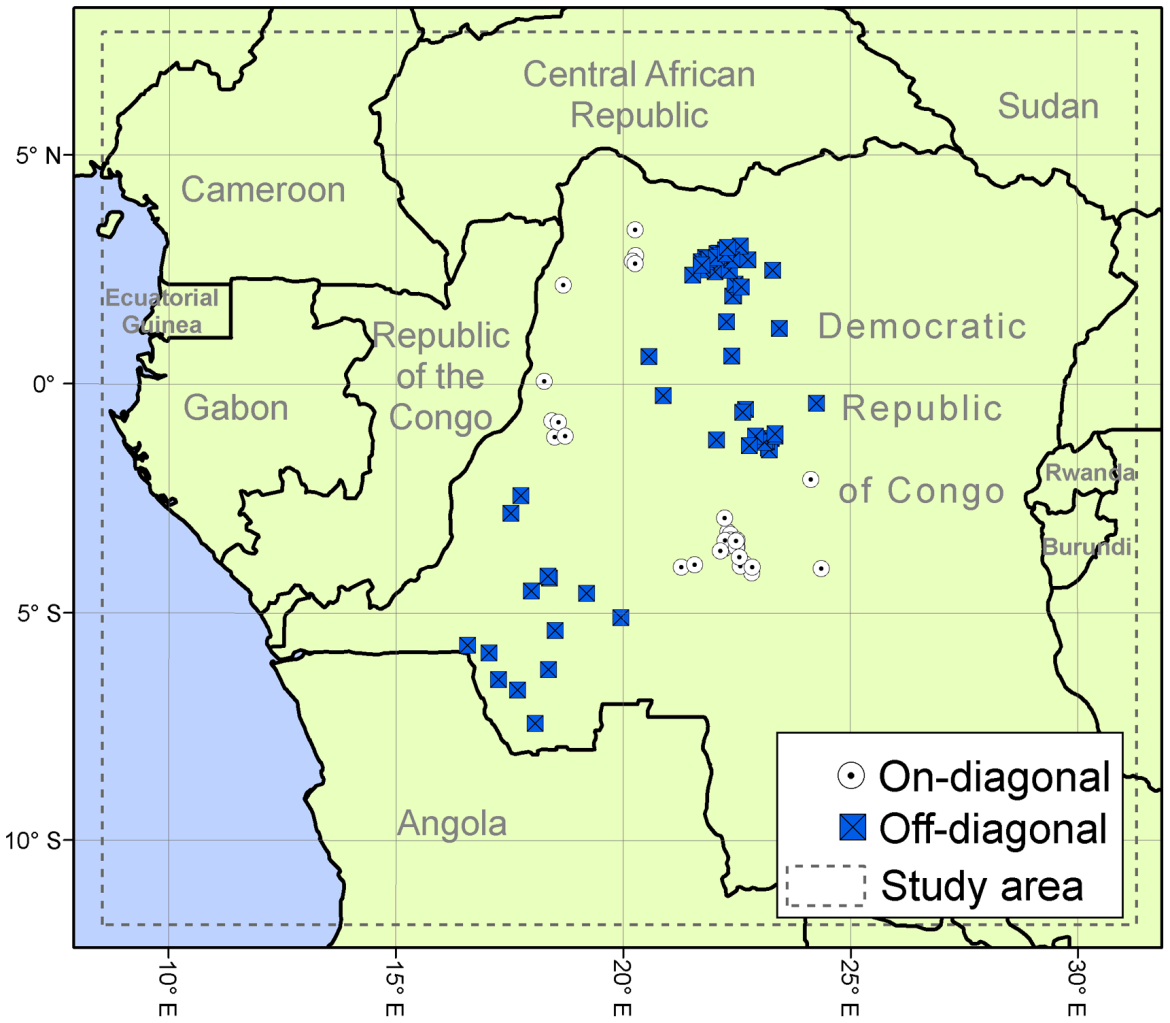
Human MPX case localities between 1982 and 1986 used for training ecological niche models. Georeferenced localities in the Democratic Republic of Congo of human MPX cases reported by WHO (n = 100), divided into two groups by median latitude and median longitude (white circles  =  on-diagonal, blue squares  =  off-diagonal). The dashed line delimits the area of interest for the present work.

Human MPX case information is almost completely based on passive surveillance with heterogeneous case report efforts across the region; as a result, we must assume that some degree of bias will be inherent in our dataset. We used the original human case reports from the WHO surveillance efforts in the DRC in 1970–1986, which were the object of extensive and intensive analyses by our research group [7], [27]. Confirmed human cases were georeferenced at the patient's residence location (representing the place where infection most likely occurred) using digital versions of 1∶250,000 Joint Operational Graphic (JOG) topographic maps for the DRC, and GEOnet Names Server (GNS; http://earth-info.nga.mil/gns.html/), in tandem with detailed case information from the original reports, and following the georeferencing procedures from MaNIS [28]; a detailed analysis of implications of different georeferencing protocols for MPX geography is provided in a separate publication [27]. Given our interest in modeling environmental conditions required for the disease to be transmitted from its wildlife reservoir to humans, cases most likely to have resulted from secondary transmission (i.e., the disease reported as acquired by contact with a sick person in the WHO case records) were eliminated from the study. In all, 100 unique localities from DRC with human cases were used (Figure 1). We assume that all of these cases correspond to the central African clade of MPXV, based on the fact that only that clade has been found in the Congo Basin to date [29], [30], [31], [32].

Recent human case data were georeferenced to provide an independent test for projections of the ENMs at a more recent time period. We followed the same methodology explained above to obtain geographic coordinates, resulting in a total of 18 unique localities where the disease was reported between 2001 and 2010 in the study area; data were drawn from the literature [33], [34] and the Institut National de Recherche Biomedicale (INRB) in DRC. The majority of localities (present and past) had a spatial uncertainty of ≤4.0 km; only 11 localities presented broader uncertainties, as high as 6.5 km. This known accuracy of georeferenced localities permitted the use of high-resolution satellite imagery (see below) in developing ENM predictions. Monkeypox surveillance in Sankuru District in 2006–2007 [5] could not be associated with precise geographic coordinates and, therefore, was not used for model evaluation; similarly, recent case reports from Central African Republic [35] were not accompanied by specific geographic references, and cases reported recently from Sudan [36], [37] likely represent central African strains exported by human activities [24], [38], such that none of these cases was included in our occurrence data sets. Although we strove for highest geographic precision for human case data, all localities used in this study (present and past) are represented by geographic coordinates for villages and a measure of uncertainty; thus, patient privacy and anonymity is not compromised since cases cannot be linked to individuals.

### Satellite imagery

To provide environmental information that covers the same time span as the human case records, we selected a dataset from Global Inventory Modelling and Mapping Studies (GIMMS; http://www.landcover.org/data/gimms) that consists of a series of biweekly maximum-value composites of Normalized Difference Vegetation Indices (NDVI) from 1982–2006 with a nominal spatial resolution of 8 km[39], which is adequate for the level of precision of the MPX dataset. NDVI is calculated based on the reflectance values of the red (λ∼0.6 µm) and near infrared (λ∼0.8 µm) bands of multispectral imagery; reflectivity in these two bands depend on the type of land cover (e.g., barren ground, cities, forest, agriculture fields, etc.) and it has been correlated with more specific characteristics of the vegetation canopy (e.g., vegetation condition, biomass, leaf area index, etc.) at the time of data collection [40], thus it is possible to track the status of the vegetation through time by analyzing the variation of this index throughout the year [41]. For these reasons, this vegetation index has been amply used for a great variety of applications including the production of global land cover classifications [42] and in many previous studies of disease distribution [43], [44] among others.

We calculated average NDVI for each month during between 1982 and 1986 (representing the earliest composites from the GIMMS dataset); then, based on these monthly data, we extracted maximum, minimum, mean, and range values for NDVI across the 12 months of each year. A similar dataset was built for 2002–2006, covering a similar time frame of 5 years, to include the most recent GIMMS composites for projections to the present. Although the environmental data for the early period do not coincide exactly in time with the earliest occurrences, satellite imagery simply does not exist prior to the period that we analyzed; however, the majority of cases come from the period covered by the imagery, which we do not believe will bias results significantly. This study focused on the Congo Basin (Figure 1, dashed-line rectangle), so all environmental layers were trimmed to this area for analysis, the area corresponding to the Atlantic Ocean is not included as part of the modeling process.

### Ecological niche models

We used the two algorithms most often applied to disease transmission questions to build ENMs for MPX transmission: a genetic algorithm (GARP) [14], [45] and a maximum entropy algorithm (Maxent) [13], [46]. These algorithms were chosen because our dataset consists of reports of human monkeypox cases that resulted from surveillance efforts that likely were not homogeneous through time across the study area; thus, although this dataset is the most complete representation of the distribution of the disease, we expect some of the bias from disease reports to remain in the dataset. Both ecological niche algorithms have been found to perform well using datasets with varying degrees of completeness [47], [48], [49]; they require presence data only, and aim to find non-random associations of disease case occurrences with the environmental conditions they present. These algorithms have been used widely by biologists and ecologists to predict species' distributions [50], [51], [52], [53]. Model transferability (i.e., prediction of distributional phenomena in other places or at other times) has been identified as an aspect of ENM that varies according to the algorithm used, and that could be the source of misleading interpretation of such models; it has been a topic for ongoing discussion in the field [48], [54]. Performance of these two algorithms (GARP and Maxent) has been tested and compared repeatedly in the last few years, with somewhat variable results, in a variety of scenarios [11], [47], [48], [55], [56], [57], [58]; hence, we used both algorithms in the present study to examine potential discrepancies and agreements of the two methodologies.

GARP builds sets of rules that describe environmental conditions associated with localities at which disease transmission events have been recorded [14], [45]. The model is developed through an iterative process of creation, evaluation, modification, and inclusion/exclusion of rules of four basic forms (bioclimatic, atomic, negated, and logistic regression rules); the algorithm stops when the optimization parameter changes by less than 1% from one iteration to the next, or when the maximum number of iterations is reached (1000). We specified that 50% of occurrence points be used by GARP for training models and the rest for internal model evaluation; we ran 100 replicate models relating human MPX cases to NDVI composites for 1982–1986. Of the initial 100 models, we selected those with the best internal performance (i.e., evaluated during the model building process) based on the ‘best subset’ consensus approach: we retained the 20% of the distribution showing lowest omission error and then the central 50% of the remaining models in terms of commission error [59]. This process resulted in selection of 10 models that were summed to identify areas of highest agreement between predictions of different replicate models.

The Maxent algorithm fits a probability distribution based on the environmental conditions at the locations where disease transmission has been recorded, in comparison with the broader set of conditions across the landscape. This algorithm estimates a model in the form of a probability distribution that is constrained to the environmental conditions present at the given locations; thus, the mean values for the environmental variables in the model will be very similar to the averages from the empirical data, the degree of similarity between the model and the data is determined by the regularization parameter (β). The maximum entropy estimate of the probability distribution is considered to be the least biased of all distributions that meet these constrains, making the model more robust regarding missing information [13], [46]. We used default settings in Maxent 3.2.1 (i.e., regularization multiplier  = 1.0, 1500 maximum iterations, 10,000 background points, convergence limit  = 10^−5^), but with a random selection of 50% of points for testing and refining the model.

First, we subjected both algorithms to the challenge of predicting human MPX in areas from which no occurrences were available for model training (spatial challenge). We divided the georeferenced WHO human cases spatially into four quadrants based on the median longitude and latitude of all cases [58] to produce two subsets (on-diagonal  =  NW + SE quadrats and off-diagonal  =  NE + SW quadrats) to break up patterns of spatial autocorrelation that could emerge from environmental variables (i.e., closer localities have similar environmental conditions). To test the ability of ENMs to capture the environmental characteristics common to MPX localities and predict other MPX localities in a different geographic area, we trained models based on each of these subsets; representing a more rigorous test than using random subsets in which localities of the testing dataset are allowed to be geographically close to a training locality [58].

We, then, performed a second challenge in which the ability of the models to predict cases in a different period of time was tested. For this second challenge, we built ENMs using all localities from 1970–1986 and environmental conditions in 1980–1986; these models were projected onto environmental conditions for 2002–2006 to be compared with the distribution of recent MPX cases.

For all models, we converted continuous model outputs into binary predictions using a modification of the Least Training Presence Threshold approach of Pearson *et al.* [57] which consists on the use of the lowest suitability value (logistic output in Maxent and model agreement in GARP) at any of the training points as the threshold value to discriminate between suitable (greater than or equal to threshold value) and unsuitable (lower than threshold value). Considering that some occurrence data may include errors since the exact locality of exposure to MPXV is not known and it could be outside of the village of residence of the reported case [58] (here estimated as *E* = 10% in light of considerable human movements from infection sites to residences), we chose as a criterion for prediction of presence the suitability value that included (100−*E*)% of the training data (i.e., we allowed the model to omit up to 10% of the training localities).

### Model evaluation

We explored the area under the curve (AUC) of the Receiver Operating Characteristic (ROC) plot as used by Elith *et al.* [11], which has been accepted broadly as the standard method to assess performance of such models. However, because the standard ROC AUC method has several weaknesses when it is applied to predictions of potential distributions [60], we also performed a variation of the original ROC method (called “modified ROC” from now on) proposed by Peterson *et al.* [58] that addresses most of these problems. The modified ROC approach has the advantage of prioritizing omission error considerations over commission error [58] and consists of selection of the domain within which the AUC is calculated via specification of an admissible omission error value (*E* again) that meets the researcher's expectations for predictive ability of the model; this step allows the method to focus on those sections of the ROC curve that are relevant to predictive performance. The AUC is calculated via the trapezoid method, and divided by the AUC value of the null expectation (i.e., the area under the line connecting 0,0 and 1,1; which represents the expected value from a random guess) over the same interval; thus, the modified ROC value departs upward from unity as the model has better predictive ability with respect to random expectations; statistical significance of this approach is calculated via bootstrapping [58], in which 1000 replicate subsamples of 50% of testing data are developed. For the spatial challenge, the ability of ENMs to predict MPX cases in a different geographic region was evaluated by determining how well a model predicts an independent set of cases that were not used to train the model; specifically, models built with the on-diagonal subset of cases were evaluated with the off-diagonal subset and vice versa. Evaluation of the predictive potential of the models for ‘future’ disease transmission events was achieved by assessing whether the model built using environmental conditions and reported cases from the 1980s could anticipate the spatial position of monkeypox cases reported in 2001–2010. One possibility is that environmental conditions and their dynamics have no effect on monkeypox transmission, resulting in no change in its geographic distribution over time; thus, we compared the predictive ability of both the 1980s model *per se* regarding 2001–2010 cases against its projection onto environmental conditions from the 2000s for prediction of the human cases in 2001–2010. We performed a Mann-Whitney *U* test based on the AUC ratio values of the respective modified ROC bootstrap analyses [58] to assess which projection predicts the distribution of recent cases better–this latter step allows us to assess whether environmental changes between the two time periods have significant explanatory power as regards monkeypox distributional patterns.

## Results

**Figure 2:**
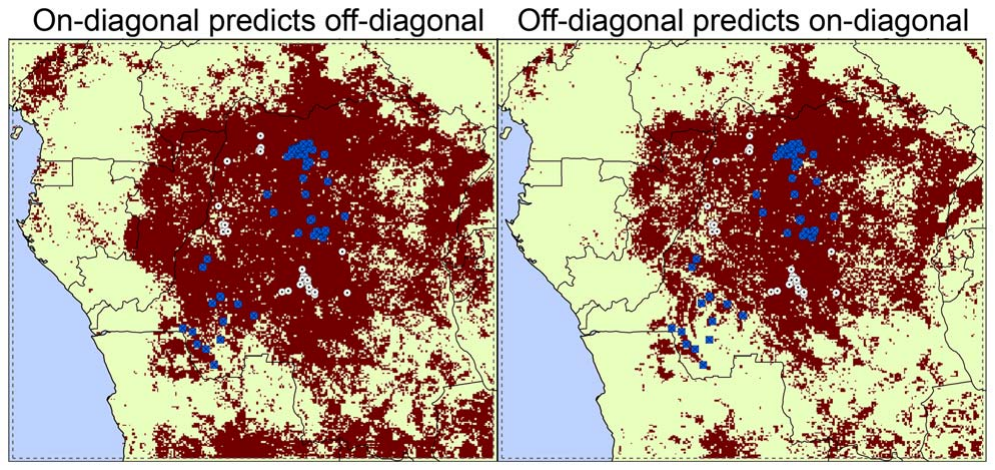
Environmentally suitable areas identified by Maxent based on independent subsets of occurrences from 1982–1986. Ecological niche model projections based on the MPX case localities of the on-diagonal (left) and the off-diagonal (right) regional subsets using environmental conditions from 1982–1986. The red area represents the suitable area identified for MPX transmission based on Maxent model; white circles  =  on-diagonal occurrences, blue squares  =  off-diagonal occurrences. Statistics associated with these tests are in Table 1.

ENMs produced by the two algorithms (GARP and Maxent) had very similar performance in our spatial testing, with that of Maxent slightly better with traditional ROC AUCs (Maxent: 0.886 and 0.877; GARP: 0.8376 and 0.8377; Table 1), but modified ROC AUCs showing mixed results (Maxent: 1.4403 and 1.3439; GARP: 1.4528 and 1.3137); both identified roughly the same areas in the central part of the Congo Basin (red areas in Figure 2), therefore, we only show results from one of them (Maxent). Via the geographic partition of the human case localities, modified ROC tests show that ENMs based on each spatial subset predicted the localities of the other subset significantly better than random, regardless of the algorithm used (all *P*<0.001; Figure 2 and Table 1). Maxent models presented higher AUC ratios than GARP models in all cases, using both traditional and modified ROC approaches (Table 1). Hence, overall, the ability of the ENMs to predict the potential distribution of MPX in broad, unsampled areas is amply confirmed.

**Table 1:** Traditional and modified ROC AUC values for ENMs built on GARP and Maxent algorithms.

MODEL TRAINING	MODEL TESTING	PARAMETER	GARP	Maxent
On-Diagonal	Off-Diagonal	Minimum	1.0914	1.2605
		Maximum	1.6151	1.7410
		Mean	1.4528	1.4403
		Standard deviation	0.0884	0.0876
		# replicates≤1	0	0
		*P*	<0.001	<0.001
		Traditional ROC AUC	0.8376	0.886
Off-Diagonal	On-Diagonal	Minimum	1.0171	1.2317
		Maximum	1.6569	1.8229
		Mean	1.3137	1.3439
		Standard deviation	0.2075	0.1637
		# replicates≤1	0	0
		*P*	<0.001	<0.001
		Traditional ROC AUC	0.8377	0.8770
1980s	2000s	Minimum	1.0014	1.1259
		Maximum	1.504	1.5011
		Mean	1.1025	1.1541
		Standard deviation	0.0992	0.0609
		# replicates≤1	0	0
		*P*	<0.001	<0.001
		Traditional ROC AUC	0.7306	0.7483
1980s projected onto 2000s conditions	2000s	Minimum	1.4170	1.4348
		Maximum	1.5431	1.7221
		Mean	1.4377	1.4449
		Standard deviation	0.2	0.326
		# replicates≤1	0	0
		*P*	<0.001	<0.001
		Traditional ROC AUC	0.7964	0.8622

Traditional and modified ROC AUC values for GARP and Maxent models trained and tested with independent spatial subsets of WHO occurrence data subsets (first two sections). Performance of models trained with data from WHO (1980s) and applied directly to recent occurrence data, or projected onto environmental conditions of the 2000s is shown in the succeeding sections. Modified ROC AUC values are reported as minimum, maximum, mean, and standard deviation of 1000 random replicates; also provided is the number of bootstrap replicates falling at or below 1.

**Figure 3:**
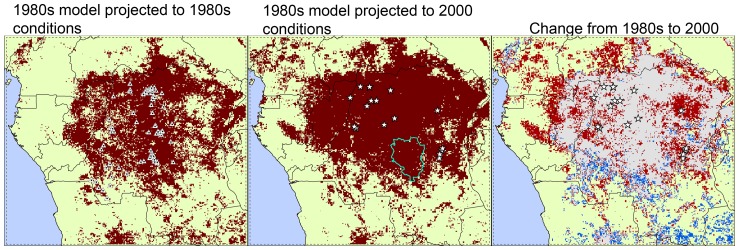
Environmentally suitable areas for MPX transmission in 1982–1986 and 2002–2006 identified by Maxent. Ecological niche model projections using human MPX case reports and environmental conditions during 1982–1986 (left) and projections onto conditions in 2002–2006 (middle; white stars  =  recent human cases); dark red represents areas with suitable environmental conditions identified by Maxent. The blue polygon in the middle map represents Sankuru District, where monkeypox surveillance was performed betweeen 2006–2007 [5]. Change in suitability for MPX transmission (i.e., difference in model-based suitability) between the 1980s and 2000s is shown in the third map (right): red  =  expansion, blue  =  reduction, and gray  =  suitable areas in both periods. Statistics associated with these tests are in Table 1.

Projection of models based on monkeypox occurrence data from WHO (1980s; triangles in Figure 3) onto environmental conditions for 2002–2006 (Figure 3, middle) anticipated the spatial distribution of recently reported MPX cases (stars in Figure 3) with high modified ROC values under both algorithms (GARP average AUC ratio = 1.30 and Maxent AUC ratio = 1.39; all *P*<0.001). In general, areas predicted as environmentally suitable for MPX transmission were broader in the projection to the 2000s than in the 1980s, with notable expansion into more northern and eastern portions of the Congo Basin. Figure 3 (right) shows ‘new’ suitable areas for MPX transmission in the 2000s (red) based on the model of 1980s and areas no longer identified as suitable when the model was projected into recent environmental conditions (blue).

Testing the hypothesis of no environmental effect on the temporal shift in the distribution of MPX cases was conducted using cases reported between 2001 and 2010. In effect, we tested whether projecting the 1980s model onto the environmental conditions of the 2000s improved the predictive ability over its projection onto 1980s conditions, with regard to recent MPX case distributions. Both algorithms predicted recent data better than random expectations (P<0.001) (Table 1, bottom). Statistical comparisons of bootstrap replicates from the two modified ROC analyses showed significant statistical differences between the accuracy of predicting the recent monkeypox occurrences using environmental data from the 2000s as opposed to the 1980s (Mann-Whitney *U* tests; GARP: *U* = 4335, P<0.001; Maxent: *U* = 3824, P<0.001), thereby supporting the view that ecologic and environmental changes during the last 30 yr have impacted the spatial distribution of monkeypox cases.

**Figure 4:**
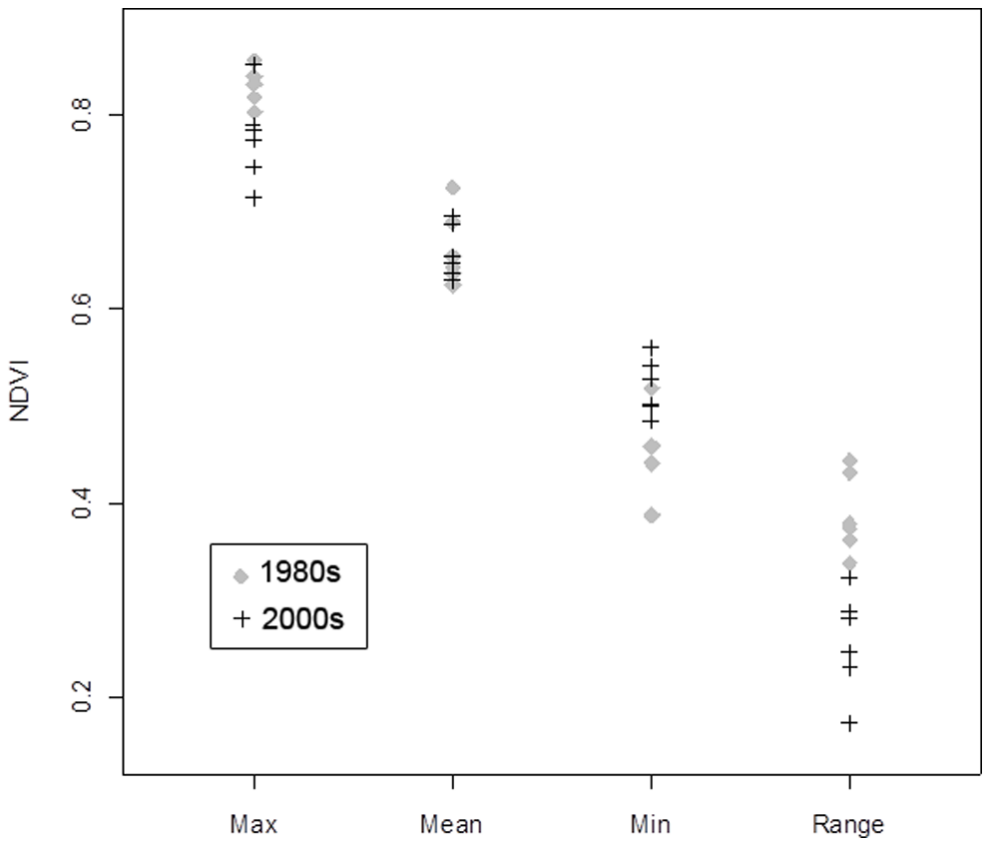
Environmental values of localities in suitable areas in 2000s but not in 1980s. Environmental conditions during the 1980s (gray diamonds) and 2000s (black crosses) at the six localities from our test dataset (2000s) that were identified as unsuitable for MPX transmission by the niche models based on occurrences in the first period (1980s) and suitable during the second period (2000s). These localities correspond to the stars in the red area of the third map (right) in Figure 3.

**Figure 5:**
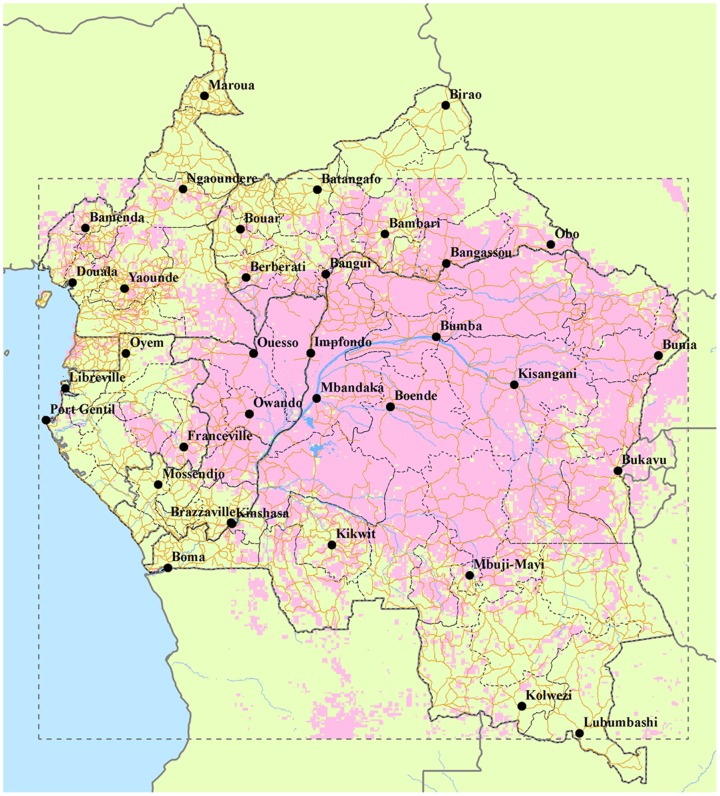
Geographic distribution of environmentally suitable areas for MPX transmission over political boundaries of the region. Environmentally suitable areas for MPX transmission based on the projection of ENMs to environmental conditions in 2000s (pink), overlaid on a political map of the study region (see Appendix for further detail). Roads are represented by orange lines and within-country administrative divisions by dashed lines.

The variables showing the highest relative contributions to the Maxent model were minimum NDVI (50%) and NDVI range (45.7%). Six localities from our testing dataset (2000s) fall in areas not identified as suitable during the 1980s but suitable in the 2000s (i.e., stars that fall in the red area of Figure 3, right); when comparing the values of our environmental variables at these localities in the 1980s with those in the 2000s, we find lower values of NDVI range and higher minimum NDVI values in the later period (Figure 4). A similar tendency for these two variables was found in Sankuru District: average minimum NDVI increased from 508.7 to 524.0, while the range decreased from 249.8 to 225.6; more of the area of this district was predicted as suitable for MPX transmission in the 2000s (98.0%) than in the 1980s (87.1%). Hence, overall, ‘suitable’ areas appear to be those presenting the least seasonal environments across the region. The percentage of area “at risk” within each time period and the change between the two time periods for each administrative unit within Congo Basin countries are shown in Appendix S1; Figure 5 shows the distribution of predicted transmission risk areas with respect to these administrative units.

## Discussion

We developed ENMs produced with the GARP and Maxent algorithms based on spatial subsets of monkeypox occurrence data from the 1980s, and demonstrated that ecological signatures in the ENMs are consistent and highly predictive of patterns of MPX transmission across space, regardless of whether data were available during model calibration. That is, models can be built based on occurrences restricted to one or more region, and used to anticipate transmission localities in other subsets. This predictive ability suggests that MPX transmission has consistent environmental correlates that can be recovered by ENM algorithms and applied in other contexts, including those that may be non-contiguous in space. This finding is consistent with results of previous studies [7], [24]. However, in the present analyses, we have extended the spatial resolution to a finer scale and to include environments at two time points, whereas earlier studies relied on climatic variables that describe broader and more static characteristics of the environment; the result of our analysis is a temporally explicit view that suggests macrogeographic shifts in transmission areas.

ENMs developed based on 1980s occurrences and projected onto environmental conditions in the 2000s predicted recent human cases significantly better than random expectations. Predictions of these time-specific projections were also significantly better than the null hypothesis of no environmental influences on shifting patterns of MPX distribution, owing to a rather dramatic northward shift in transmission areas across the Congo Basin. This evidence supports the idea that spatial shifts in transmission suitability are linked to particular environmental conditions that affect elements of the natural transmission cycle directly (e.g., distribution of the natural reservoir, transmission rate, or exposure of humans to reservoirs).

The variables with the highest contribution to the Maxent model (minimum NDVI and range) suggest that environments with low variation through the year and that maintain certain level of greenness are necessary for MPX transmission (i.e., rainforest); although this association is not novel [4], [26], it is important to note that suitable environments cover a broader area in the 2000s than 20 years before, suggesting an increase of the spatial footprint of the MPX risk area. This increase is particularly marked in the eastern and northern parts of the study area, while the tendency in the southern part is toward reduction of suitable areas (blue in Figure 3, right). Shifts in the geographic position of the suitable area, suggest new areas where disease surveillance would be useful owing to possible new exposure of human populations to MPXV transmission in such areas.

Details of how is MPXV maintained in nature and transmitted from wildlife to humans are currently unknown; however, although several mammal species (e.g., rodents, marsupials, primates, etc.) have been identified to be susceptible to experimental or natural infection with this virus, the host(s) involved in its natural cycle reminds undetermined [61]. Rodents have been identified as potential reservoirs of monkeypox because they have rapid population turnover that could facilitate perpetuation of the virus; other members of the genus *Orthopoxvirus* are associated with species of rodents [62], [63]; and the only MPXV isolate from a wild animal was obtained from a squirrel of the genus *Funisciurus* [64]. Additionally, some species of rodents susceptible to MPXV infection (e.g., species of the genera *Funisciurus*, *Heliosciurus* and *Cricetomys*) can be found at the margins between human communities and rainforest in DRC [8], [9]. Studies on the rodent hosts of other zoonoses have shown that products from satellite imagery such as NDVI are associated with their population densities and virus prevalence in them [65], [66]. Similarly, the host(s) of MPXV could be responding to changes in environmental variables associated with NDVI (vegetation health, phenology, biomass, etc.) with variations in their geographic distributions and/or populations. These variations in the host could directly affect MPXV prevalence in their populations and, therefore, the possibility of human infection.

A recent paper reported increasing numbers of human MPX cases associated with increasing proportions of the population that have not received the smallpox vaccine and that are, therefore, more susceptible to MPX infection [4], [6], a conclusion with which we do not necessarily disagree. However, the proportion of vaccinated vs. unvaccinated human populations is not the only factor that has changed in the last 30–40 years; for example, land use has changed dramatically in several areas in DRC from natural vegetation cover to urban areas, grazed areas, or agricultural fields [67]. These changes and the patterns of interaction between humans and nature could favor human contact with MPXV reservoirs or hosts, which could increase the probability of transmission of the virus. Our study identified a higher proportion of Sankuru District's area as suitable for monkeypox transmission in the second period; this could represent higher suitability for MPXV or its reservoir(s) and a higher chance of exposure of people to them. Because of the limited information about the natural history of MPXV, it is very difficult to estimate people's level of exposure to the virus in the past or if it has changed since 1980's, but it is equally hard to assume that the level of exposure has remained the same throughout the years and the only variable that has changed is the proportion of the susceptible people in the population via waning vaccination protection.

Transmission of zoonotic diseases depends on interactions between reservoir, pathogen, and human, but several factors also play important roles on determining whether such interactions can result in disease transmission: environmental conditions (as shown here), susceptibility of humans to infection [4], prevalence of the pathogen, level of interaction between humans and reservoirs (e.g., wildlife vs. domesticated animals; forest species vs. peridomestic species), and route of transmission (i.e., direct or indirect contact, aerosol, vector-borne). In the case of MPX many of these factors are been currently studied at the appropriate scales (e.g., landscape, community), but the association of shifting suitable MPX transmission areas with human MPX cases over the past few decades is perhaps indicative of a broader-scale causation, beyond the changing proportion of immunologically naïve individuals in the population; thus, environmental dimensions should not be neglected.

## Supporting Information

Appendix S1 Coverage of suitable environments for MPX transmission per administrative unit. Area identified in this study as at risk for MPX transmission for each administrative unit of the Democratic Republic of Congo, the Republic of the Congo, Central African Republic, Gabon, and Cameroon. The percentage of that area identified as suitable for monkeypox transmission by each algorithm in each time period and the percent change in suitable area from the 1980s to the 2000s are both provided.
(DOCX)
