# Supplementary material for: Mapping Monkeypox Transmission Risk through Time and Space in the Congo Basin
Source: PLoS One. 2013 Sep 5;8(9):e74816. doi: 10.1371/journal.pone.0074816 (PMC3764067; doi:10.1371/journal.pone.0074816)
Supplement: Appendix S1 — Coverage of suitable environments for MPX transmission per administrative unit. Area identified in this study as at risk for MPX transmission for each administrative unit of the Democratic Republic of Congo, the Republic of the Congo, Central African Republic, Gabon, and Cameroon. The percentage of that area identified as suitable for monkeypox transmission by each algorithm in each time period and the percent change in suitable area from the 1980s to the 2000s are both provided. (DOCX) [file pone.0074816.s001.docx]

**Appendix S1**

|  |  |  | **Maxent** | | **GARP** | | **1980s to 2000s** | |
| --- | --- | --- | --- | --- | --- | --- | --- | --- |
| **Country** | **District/department /prefecture/province**  **/region** | **Area included (1000 km^2^)** | **1980s (%)** | **2000s (%)** | **1980s (%)** | **2000s (%)** | **Maxent**  **% change** | **GARP**  **% change** |
| Democratic | Bas-Uele | 147.94 | 78.85 | 92.65 | 75.06 | 90.93 | 13.80 | 15.87 |
| Republic | Équateur | 103.46 | 67.84 | 97.79 | 63.53 | 96.64 | 29.95 | 33.11 |
| of Congo | Haut-Katanga | 101.64 | 22.06 | 5.01 | 18.09 | 3.69 | -17.04 | -14.40 |
|  | Haut-Lomami | 110.03 | 15.12 | 14.45 | 12.51 | 12.91 | -0.67 | 0.39 |
|  | Haut-Uele | 91.75 | 44.61 | 76.78 | 39.50 | 71.70 | 32.17 | 32.20 |
|  | Ituri | 64.69 | 60.29 | 89.65 | 58.09 | 87.73 | 29.36 | 29.64 |
|  | Kasai | 97.10 | 59.99 | 64.01 | 54.69 | 56.88 | 4.02 | 2.19 |
|  | Kasai oriental | 10.55 | 43.75 | 37.50 | 29.57 | 16.59 | -6.25 | -12.98 |
|  | Kinshasa | 10.98 | 9.47 | 17.55 | 4.85 | 5.31 | 8.08 | 0.46 |
|  | Kongo central | 54.04 | 7.27 | 11.31 | 6.19 | 7.65 | 4.04 | 1.45 |
|  | Kwango | 89.01 | 27.41 | 34.25 | 23.30 | 24.90 | 6.84 | 1.60 |
|  | Kwilu | 80.99 | 54.92 | 45.55 | 37.23 | 24.83 | -9.36 | -12.40 |
|  | Lomami | 52.82 | 37.30 | 45.03 | 33.51 | 35.38 | 7.73 | 1.87 |
|  | Lualaba | 123.27 | 22.96 | 15.53 | 19.30 | 12.63 | -7.43 | -6.67 |
|  | Lulua | 56.02 | 66.64 | 57.63 | 59.08 | 46.27 | -9.01 | -12.81 |
|  | Mai-Ndombe | 126.00 | 77.20 | 92.27 | 67.68 | 87.48 | 15.07 | 19.80 |
|  | Maniema | 128.72 | 50.85 | 79.96 | 44.60 | 76.81 | 29.12 | 32.21 |
|  | Mongala | 57.46 | 94.09 | 99.87 | 91.97 | 99.21 | 5.78 | 7.24 |
|  | Nord-Kivu | 59.82 | 71.81 | 81.81 | 67.74 | 75.88 | 10.00 | 8.14 |
|  | Nord-Ubangi | 56.07 | 79.92 | 92.09 | 78.20 | 87.52 | 12.17 | 9.32 |
|  | Sankuru | 105.29 | 87.14 | 97.59 | 81.14 | 91.86 | 10.45 | 10.72 |
|  | Sud-Kivu | 62.00 | 50.06 | 68.88 | 44.29 | 64.05 | 18.81 | 19.75 |
|  | Sud-Ubangi | 52.31 | 82.06 | 96.27 | 77.41 | 94.18 | 14.20 | 16.77 |
|  | Tanganyika | 121.62 | 8.51 | 18.68 | 7.01 | 17.10 | 10.18 | 10.09 |
|  | Tshopo | 196.83 | 59.19 | 96.06 | 55.27 | 94.74 | 36.87 | 39.47 |
|  | Tshuapa | 134.73 | 91.76 | 99.85 | 88.24 | 99.70 | 8.09 | 11.46 |
| Republic | Bouenza | 12.25 | 0.00 | 0.00 | 0.00 | 0.00 | 0.00 | 0.00 |
| of the | Brazzaville | 0.28 | 0.00 | 0.00 | 0.00 | 0.00 | 0.00 | 0.00 |
| Congo | Cuvette | 39.76 | 72.51 | 97.96 | 67.60 | 95.09 | 25.45 | 27.49 |
|  | Cuvette-Ouest | 29.59 | 47.56 | 95.03 | 34.02 | 82.52 | 47.47 | 48.50 |
|  | Kouilou | 13.49 | 0.00 | 0.00 | 0.00 | 0.00 | 0.00 | 0.00 |
|  | Lékoumou | 21.22 | 0.00 | 8.84 | 0.00 | 2.15 | 8.84 | 2.15 |
|  | Likouala | 66.54 | 76.03 | 98.48 | 70.27 | 97.52 | 22.45 | 27.25 |
|  | Niari | 25.56 | 0.00 | 1.39 | 0.00 | 0.00 | 1.39 | 0.00 |
|  | Plateaux | 39.76 | 73.85 | 86.16 | 53.70 | 56.44 | 12.31 | 2.74 |
|  | Pool | 35.37 | 7.24 | 15.48 | 4.01 | 5.66 | 8.24 | 1.65 |
|  | Sangha | 57.79 | 46.29 | 88.15 | 43.26 | 86.22 | 41.86 | 42.96 |
| Central | Bamingui-Bangora | 8.55 | 0.00 | 6.53 | 0.00 | 4.75 | 6.53 | 4.75 |
| African | Bangui | 3.04 | 40.83 | 86.67 | 34.17 | 70.00 | 45.83 | 35.83 |
| Republic | Basse-Kotto | 16.31 | 37.01 | 62.67 | 35.93 | 54.74 | 25.66 | 18.82 |
|  | Haut-Mbomou | 52.82 | 21.17 | 42.49 | 17.43 | 37.45 | 21.32 | 20.02 |
|  | Haute-Kotto | 51.48 | 32.46 | 57.59 | 28.52 | 50.49 | 25.12 | 21.97 |
|  | Kémo | 16.38 | 7.12 | 67.34 | 5.57 | 45.20 | 60.22 | 39.63 |
|  | Lobaye | 9.76 | 34.81 | 45.45 | 33.25 | 43.38 | 10.65 | 10.13 |
|  | Mambéré-Kadéï | 29.75 | 14.07 | 31.20 | 12.36 | 23.36 | 17.14 | 11.00 |
|  | Mbomou | 60.38 | 68.92 | 85.13 | 67.49 | 83.24 | 16.21 | 15.75 |
|  | Nana-Grébizi | 17.04 | 4.32 | 52.53 | 4.02 | 41.67 | 48.21 | 37.65 |
|  | Nana-Mambéré | 27.16 | 1.68 | 14.01 | 1.68 | 10.46 | 12.32 | 8.78 |
|  | Ombella-M'Poko | 37.25 | 22.26 | 46.77 | 19.88 | 36.83 | 24.51 | 16.95 |
|  | Ouaka | 49.09 | 5.89 | 47.52 | 3.72 | 36.26 | 41.63 | 32.54 |
|  | Ouham | 45.52 | 4.35 | 20.61 | 2.95 | 14.54 | 16.27 | 11.59 |
|  | Ouham-Pendé | 31.98 | 0.79 | 7.14 | 0.56 | 5.00 | 6.34 | 4.44 |
|  | Vakaga | Outside of study area | | | | | | |
|  | Sangha-Mbaéré | 18.33 | 74.69 | 96.13 | 69.85 | 93.64 | 21.44 | 23.79 |
| Gabon | Estuaire | 19.60 | 0.91 | 2.33 | 0.26 | 0.39 | 1.42 | 0.13 |
|  | Haut-Ogooue | 33.83 | 23.91 | 61.24 | 2.40 | 22.94 | 37.33 | 20.54 |
|  | Moyen-Ogooue | 16.53 | 0.31 | 14.42 | 0.00 | 1.23 | 14.11 | 1.23 |
|  | Ngounie | 38.57 | 0.00 | 4.14 | 0.00 | 0.79 | 4.14 | 0.79 |
|  | Nyanga | 22.34 | 0.45 | 0.34 | 0.00 | 0.00 | -0.11 | 0.00 |
|  | Ogooue-Ivindo | 43.49 | 0.23 | 31.49 | 0.29 | 20.76 | 31.25 | 20.47 |
|  | Ogooue-Lolo | 28.98 | 4.72 | 55.29 | 3.50 | 38.50 | 50.57 | 35.00 |
|  | Ogooue-Maritime | 23.76 | 0.75 | 7.15 | 0.00 | 0.11 | 6.40 | 0.11 |
|  | Woleu-Ntem | 37.58 | 0.00 | 8.57 | 0.00 | 2.97 | 8.57 | 2.97 |
| Cameroon | Adamaoua | 60.63 | 2.84 | 25.43 | 2.22 | 23.80 | 22.58 | 21.58 |
|  | Centre | 68.57 | 0.52 | 35.32 | 0.33 | 32.10 | 34.80 | 31.77 |
|  | Est | 108.84 | 12.14 | 46.60 | 9.72 | 45.01 | 34.46 | 35.30 |
|  | Extreme-Nord | Outside of study area | | | | | | |
|  | Littoral | 20.34 | 2.74 | 18.45 | 2.12 | 12.84 | 15.71 | 10.72 |
|  | Nord | 9.53 | 0.00 | 2.13 | 0.00 | 1.60 | 2.13 | 1.60 |
|  | Nord-Ouest | 17.40 | 35.71 | 34.55 | 30.47 | 29.59 | -1.17 | -0.87 |
|  | Ouest | 14.00 | 20.29 | 38.95 | 13.77 | 35.69 | 18.66 | 21.92 |
|  | Sud | 47.47 | 0.37 | 11.97 | 0.37 | 3.90 | 11.59 | 3.53 |
|  | Sud-Ouest | 24.72 | 21.85 | 41.03 | 14.97 | 26.46 | 19.18 | 11.49 |
